# Novel natural inhibitors targeting B-RAF(V600E) by computational study

**DOI:** 10.1080/21655979.2021.1943113

**Published:** 2021-07-12

**Authors:** Bo Wu, Zhiyun Zhang, Gaojing Dou, Xiaye Lv, Junliang Ge, Hongyu Wang, Haoqun Xie, Dong Zhu

**Affiliations:** a Department of Orthopaedics, the First Bethune Hospital of Jilin University, Street Xinmin 71, Changchun, China; b Clinical College, Jilin University, Street Xinmin 126, Changchun, China; c Department of Breast Surgery, the First Bethune Hospital of Jilin University, Street Xinmin 71, Changchun, China; d Department of Hematology, the First Clinical Medical School of Lanzhou University, No.1, Donggangxi Rd, Chengguan District, Lanzhou, Gansu

**Keywords:** B-raf(v600e), vemurafenib, drug treatment, discovery studio, virtual screening

## Abstract

The aim of this research was to screen the ZINC15 database to select lead compounds and drug candidates which can inhibit B-RAF (V600E). In order to identify drugs potentially inhibited B-RAF (V600E), numerous modules of Discovery Studio 4.5 were employed. Structure-based screening using LibDock was carried out followed by ADME (absorption, distribution, metabolism, excretion) and toxicity prediction. CDOCKER was performed to demonstrate the binding affinity and mechanism between ligands and B-RAF(V600E). To evaluate whether ligand-receptor complexes were stable, molecular dynamics were employed. Two novel natural compounds (ZINC000100168592 and ZINC000049784088) from ZINC15 database were found binding to B-RAF(V600E) with more favorable interaction energy in comparison with the reference drug Vemurafenib. Also, they were predicted with less ames mutagenicity, rodent carcinogenicity, non-developmental toxic potential and tolerance to cytochrome P450 2D6 (CYP2D6). The molecular dynamics simulation analysis indicated that the compound-B-RAF(V600E) complexes had more favorable potential energy compared with Vemurafenib and they can exist in natural environments stably. The result of this study shows that ZINC000100168592 and ZINC000049784088 are ideal leading potential compounds to inhibit B-RAF(V600E). The findings of this study and these selected drug candidates greatly contributed to the medication design and improvement of B-RAF(V600E) and other proteins.

## Graphical abstract

**Figure:**
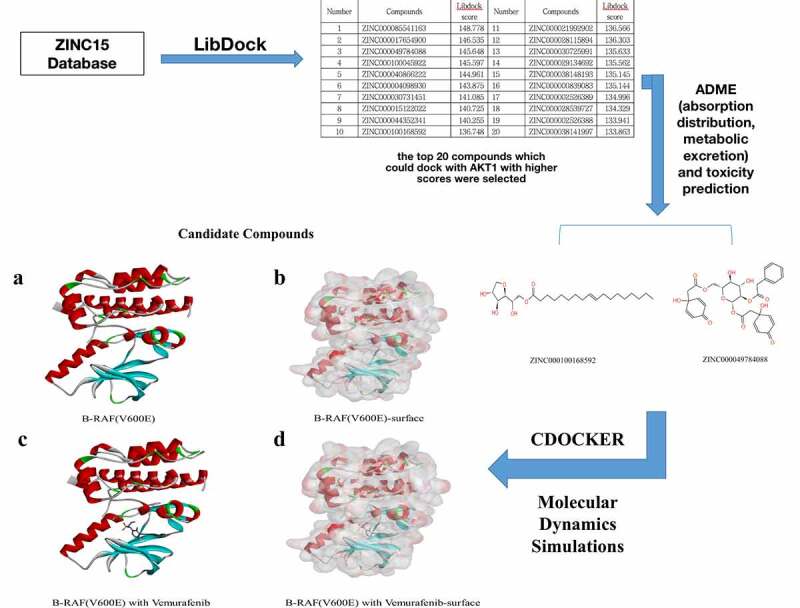

## Introduction

Melanoma is a kind of skin cancer of great aggressiveness and it has significant morbidity and mortality [1,2]. It has been previously observed that the malignant proliferation and transformation of melanocytes appeared in many parts of the body (skin, uvea, gastrointestinal mucosa, genitourinary mucosa, and CNS) [3]. In spite of its low proportion (1%) of all skin cancers, it became the leading cause of death in this group [4,5]. In different stages, the survival rate for patients with melonoma varies, the five-year survival of stages I and II is 98%, stage III is 64%, stage IV is merely 23% [6]. Surgery is the treatment of early-stage patients, which is highly curable [7]. Before 2011, chemotherapy was the only option available for many patients with advanced and unresectable melanoma [8]. Recent years, there have been numerous adjuvant approvals such as immunotherapy and targeted therapy approaches [7,8]. However, the prognosis of the patients is not good. Recently published data from the 5-year pooled analysis of COMBI(Combination therapy)-d and COMBI(Combination therapy)-v trials of dabrafenib and trametinib report an overall survival rate of 34% [9]. One of the reasons for the poor prognosis is that certain enzymes in the body prevent cancer drugs killing tumor cells through certain mechanisms.

The RAF family comprises A-RAF, B-RAF, and C-RAF [10]. In contrast to C-RAF and A-RAF, the basal kinase activity of B-RAF is the highest, and B-RAF is the most frequently activated by somatic mutations in malignancies (approximately 7%-9%) [11–13]. Besides, B-RAF is the most potent activator of MEK (Mitogen-activated protein kinase) in the RAS-MAPK signaling pathway which plays an important role in cell differentiation, proliferation and survival [10,11]. Furthermore, nearly 80% of the mutations of B-RAF observed is B-RAF(V600E) mutation [12]. Thus, B-RAF(V600E) plays a vital role in cancer targeted therapy.

Through hydrophobic interactions between the activation segment and the negatively charged regulatory region of B-RAF, which block the docking of ATP and the substrate, B-RAF keeps its inactive conformation [13]. When the two key sites of the activation segment (T599 and S602 for B-RAF) phosphorylate, negative charge will be provided. Given that the negative charge disrupts these hydrophobic interactions, the protein will be enzymatically active [13,14]. Study indicates that V600E mutation can mimic the phosphorylation at T599/S602 (phosphomimetic mutation), which can provide negative charge and have the B-RAF activated. As a result, B-RAF(V600E) has potent oncogenic activity [3,13,14].

In summary, the B-RAF(V600E) mutation can activate B-RAF which is the most potent activator of MEK (Mitogen-activated protein kinase) in the RAS-RAF-MEK-ERK signaling pathway. When the pathway is continuously and abnormally activated, cell proliferation, cell differentiation and cell viability will be abnormal, which may lead to the occurrence of tumor. B-RAF(V600E) mutations are present in hairy-cell leukemia, cutaneous melanoma, thyroid carcinomas and, less commonly, in ovarian, colon, lung, and other malignancies [15]. Hence, effective complexes targeting B-RAF(V600E) needed to be developed. Vemurafenib is a kind of drug of great selectivity targeting B-RAF(V600E). Because of its improving therapeutic effect on melanoma patients with B-RAF(V600E) mutations, rapid tumor response, low toxicity profile and tolerance, it quickly received the approval of FDA in 2011 [16]. It inhibits B-RAF kinase to decrease the downstream activation of MAPK pathway, resulting in the cell cycle arrest and apoptosis of B-RAF(V600E) mutation cells increased [17]. Although the efficacy of vemurafenib has been confirmed clinically, its durability is still a serious problem since many patients develop resistance to it after 6–8 months [18]. Hence, this study aimed to screen natural compounds from natural drugs that are more effective in inhibiting B-RAF(V600E).

Natural products, as lead compounds, can be transformed into new drugs through appropriate structural modification, which is an important source of new drug research in pharmaceutical industry. Paclitaxel and fungal metabolites and/or their analogues were good examples of natural products used in anti-tumor therapy [19,20]. Paclitaxel has been widely used in the treatment of breast cancer, and a number of fungal metabolites and/or their analogues such as anguidine, aphidicolin, fumagillin, illudins, irofulven, rhizoxin, wort-mannin, plinabulin (NPI-2358, a semisynthetic analogue of Phenyl-ahistin) and sonolisib (PX-866, a synthetic analogue of wortmannin) have progressed to various stages of cancer clinical trials [21]. In recent years, there has been an increasing interest in developing drugs targeting B-RAF(V600E). This prospective study was designed to screen natural compounds to verify potential inhibitors of B-RAF(V600E).

Recently, more and more emerging technologies have been integrated into the research of cancer detection and treatment [22,23]. In order to find new potential B-RAF(V600E) inhibitor, our study carried out a virtual screening against the Natural Products database (NP) in the ZINC database by using multiple modules of Discovery Studio 4.5. We analyzed the ADME (absorption, distribution, metabolism, excretion) and toxicity properties of molecules. By docking, we also analyzed binding modes and interactions between potential compounds and B-RAF(V600E). To assess if their binding interactions are stable, we performed a molecular dynamics simulation. This study lays groundwork for future clinical trials of these identified compounds and these identified novel natural compounds with structural modifications can be potential contributors for leads in further rational drug design for targeting B-RAF(V600E). The drug screened in this study is the first to be used in the inhibitory study of B-RAF(V600E), which will generate fresh insight into finding potential compounds to inhibit B-RAF (V600E). Besides, the method we used can also be helpful for the research of other drugs.

## Methods

### Discovery studio software and Ligand library

Discovery Studio 4.5 software (BIOVIA, San Diego, California, USA) is a suite of software for simulating small molecule and macromolecule systems; it is a new generation of molecular modeling and environmental simulation software for the life sciences field [22]. It aims to provide protein modeling, optimization, and drug design tools by applying protein structure and structural biologic computation. Numerous lead compounds and drug candidates were identified and refined through this method. LibDock module of Discovery Studio was employed for virtual screening; CDOCKER module was used for docking study; and ADME module was analyzed for pharmacologic properties. The Natural Products database in the ZINC15 database was selected to screen B-RAF(V600E) inhibitors. The ZINC15 database is a free database of commercially available compounds provided by the Irwin and Shoichet Laboratories, Department of Pharmaceutical Chemistry, University of California, San Francisco (San Francisco, California, USA) [24]

### Use LibDock for structure-based virtual filtering

**Figure 1.**
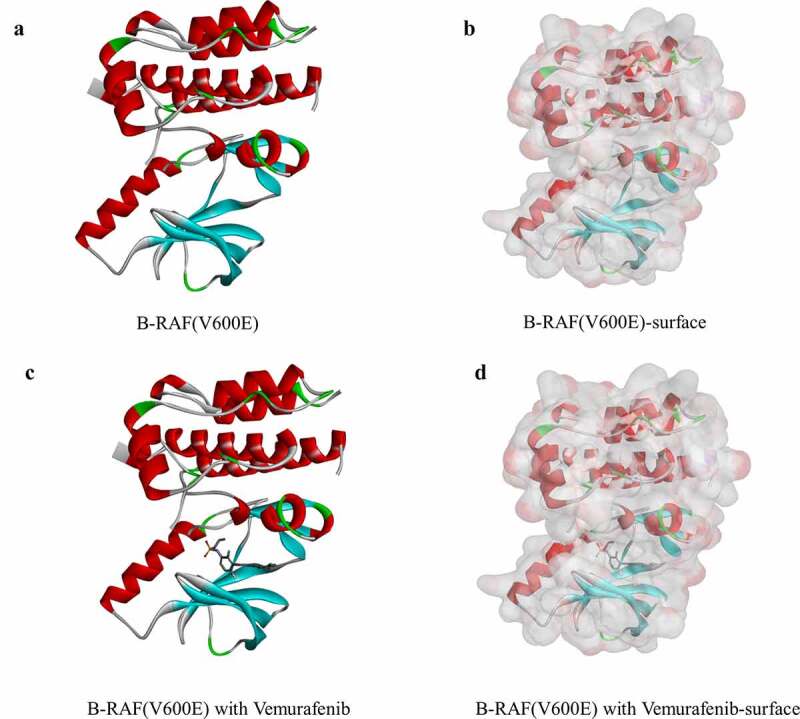
Molecular structure of B-RAF(V600E). (a) Initial molecular structure. (b) Surface of binding area added. Blue represents positive charge, and red represents negative charge. (c) Vemurafenib of binding area added

Ligand-binding pocket region of B-RAF(V600E) was selected as the binding site to screen compounds that could potentially inhibit B-RAF(V600E). Virtual screening was carried out using the LibDock module of Discovery Studio 4.5 [25]. LibDock is a rigid-based docking module. It calculates hotspots for the protein using a grid placed into the binding site and polar and a nonpolar probe. The hotspots are further used to align the ligands to form favorable interactions. The Smart Minimizer algorithm and CHARMM force field (Harvard University, Cambridge, Massachusetts, USA) were performed for ligand minimization [26]. After minimization, all ligand poses were ranked based on the ligands score. The 2.0 Å crystal structure of human B-RAF(V600E) and the inhibitor Vemurafenib were downloaded from the Protein Data Bank and imported to the working circumstance of LibDock. The molecule structure of B-RAF(V600E) was shown in Figure 1. The protein was prepared by removing crystal water and other heteroatoms around it, followed by addition of hydrogen, protonation, ionization, and energy minimization. The CHARMM force field and the Smart Minimizer algorithm were applied for energy minimization. The minimization performed 2000 steps with a root mean square gradient tolerance of 12.277, and the final root mean square gradient was 0.690. The prepared protein was employed to define the binding site. Using the ligands B-RAF(V600E) binding position, the active site for docking was generated. Virtual screening was performed by docking all the prepared ligands at the defined active site using LibDock. Based on the LibDock score, all the docked poses were ranked and grouped, and all compounds were ranked according to the LibDock score.

### ADME (Absorption, Distribution, Metabolism, and Excretion) and Toxicity Prediction

The ADME module of Discovery Studio 4.5 was employed to calculate absorption, distribution, metabolism, and excretion (ADME) of selected compounds, including their aqueous solubility, blood–brain barrier penetration, cytochrome P-450 2D6 (CYP2D6) inhibition, hepatotoxicity, human intestinal absorption and plasma protein binding level [27]. TOPKAT module of Discovery Studio 4.5 was employed to calculate the toxicity and other properties of all the potential compounds, such as U.S. National Toxicology Program rodent carcinogenicity, Ames mutagenicity, developmental toxicity potential, and rat oral median lethal dose (LD50) and chronic oral lowest observed adverse effect level (LOAEL). These pharmacologic properties were fully considered when selecting proper drug candidates for B-RAF(V600E)

### Analysis of ligand binding and ligand pharmacophore

On the basis of the CHARMM force field used for both receptors and ligands, CDOCKER, a molecular docking module of the software, was employed for the study of docking, providing docking results with high precision [28]. During the docking process, the receptor is held rigid, while ligands are allowed to flex, besides, the interaction energy and the CHARMM energy of the complexes can be calculated. Crystal structure of B-RAF(V600E) was obtained from the protein data bank. Our study removed crystalline water molecules in semi-flexible and rigid docking, which may result in the conformation of the receptor ligand complex to be affected by the fixed water molecules. Then, the protein was hydrogenated. For the purpose of verifying if the combination was reliable, Vemurafenib was removed from the binding site of B-RAF(V600E) and re-docked into it in order to compare the root-mean-square deviation (RMSD) of these two conformations. The binding site sphere of B-RAF(V600E) was defined as the regions that come within 5-Å radius from the geometric centroid of the ligand Vemurafenib. The structures of identified hits were prepared and docked into the binding pocket of B-RAF(V600E). Based on the interaction energy CODOCKER module provided, different positions of each ligand-B-RAF(V600E) complex were analyzed. 3D-QSAR, one of modules of Discovery Studio 4.5, was employed to generate the pharmacophores of the compounds and their pharmacophores were displayed.

### Molecular Dynamics Simulation

The best binding conformations of the ligand-B-RAF(V600E) complexes among the poses predicted by the molecule docking program were submitted to MD simulation using Discovery Studio 4.5 [29]. The ligand-receptor complex was put into an orthorhombic box and solvated with an explicit periodic boundary solvation water model. In order to simulate the physiological environment, sodium chloride was added to the system with an ionic strength of 0.145. Then the system was subjected to the CHARMM force field which was used for ligand parameterization based on analogy. For the system, the following simulation protocols were applied: 1000 steps of minimization by steepest descent and conjugate gradient; 5ps-equilibration simulations in temperature of 300 K (slowly driven from initial temperature of 50 K for 2ps) and normal pressure ensemble; 25ps-MD simulation (production module) under NPT (normal pressure and temperature). The Particle Mesh Ewald (PME) algorithm was used to calculate long-range electrostatics, and the linear constraint solver (LINCS) algorithm was adapted to fix all bonds involving hydrogen. With initial complex setting as a reference, a trajectory was determined for root mean-square deviation(RMSD), potential energy, and structural characteristics through the Discovery Studio 4.5 analysis trajectory protocol in Discovery Studio 4.5.

## Results

### Virtual Screening of Natural Products Database Against B-RAF(V600E)

Ligand-binding pocket was a critical regulatory site of B-RAF(V600E). When the ligand pocket was connected with some inhibitor, V600E could not give B-RAF negative charges to break the hydrogen interactions, thus preventing V600E from activating B-RAF, therefore, this pocket region was selected as a reference site. A total of 17,799 purchasable natural named product molecules were taken from the ZINC15 database. Molecular structure of B-RAF(V600E) was selected as the receptor protein. Vemurafenib, one of B-RAF(V600E) inhibitors, was chosen as a reference compound to evaluate the binding ability of other compounds. 7763 compounds were identified to bind with STING stably by the LibDock algorithm. The top 20 compounds with higher LibDock scores than were listed in Table 1.

**Table 1.** Top 20 ranked compounds with higher libdock scores than Vemurafenib

Number	Compounds	Libdock score	Number	Compounds	Libdock score
1	ZINC000085541163	148.778	11	ZINC000021992902	136.566
2	ZINC000017654900	146.535	12	ZINC000028115894	136.303
3	ZINC000049784088	145.648	13	ZINC000030725991	135.633
4	ZINC000100045922	145.597	14	ZINC000029134692	135.562
5	ZINC000040866222	144.961	15	ZINC000038148193	135.145
6	ZINC000004098930	143.875	16	ZINC000000839083	135.144
7	ZINC000030731451	141.085	17	ZINC000002526389	134.996
8	ZINC000015122022	140.725	18	ZINC000028539727	134.329
9	ZINC000044352341	140.255	19	ZINC000002526388	133.941
10	ZINC000100168592	136.748	20	ZINC000038141997	133.863

### ADME and Toxicity Prediction

Pharmacological properties of all selected ligands and Vemurafenib were first predicted by ADME module of Discovery Studio 4.5, including brain/blood barrier (BBB), human intestinal absorption, aqueous solubility, cytochrome P450 2D6 (CYP2D6) binding, hepatotoxicity and plasma protein binding properties (PPB) (Table 2). The aqueous solubility prediction (defined in water at 25°C) indicated that eight compounds were soluble in water. For human intestinal absorption, five compounds had a good absorption level and two compounds and Vemurafenib had a moderate absorption level. Eight compounds and Vemurafenib were found to be highly bound with plasma protein and the rest were just opposite. Seventeen compounds and Vemurafenib were predicted to be non-inhibitors of cytochrome P450 2D6 (CYP2D6), which was one of the important enzymes involved in drug metabolism. For hepatotoxicity, 11 compounds were predicted as nontoxic, which was similar to Vemurafenib

**Table 2.** ADME (Adsorption, Distribution, Metabolism, Excretion) properties of compounds

Number	Compounds	Solubility Level^a^	BBB level^b^	CYP2D6^c^	Hepatotoxicity^d^	Absorption Level^e^	PPB Level^f^
1	ZINC000085541163	2	4	0	0	2	0
2	ZINC000017654900	2	4	0	1	2	0
3	ZINC000049784088	4	4	0	0	3	0
4	ZINC000100045922	0	4	0	0	3	1
5	ZINC000040866222	3	4	0	0	2	0
6	ZINC000004098930	3	4	0	1	2	0
7	ZINC000030731451	3	4	0	0	0	0
8	ZINC000015122022	2	4	1	0	2	1
9	ZINC000044352341	4	4	0	0	3	0
10	ZINC000100168592	3	4	0	0	1	1
11	ZINC000021992902	3	4	0	0	1	0
12	ZINC000028115894	0	4	0	1	3	0
13	ZINC000030725991	0	4	0	1	2	0
14	ZINC000029134692	2	1	0	0	0	1
15	ZINC000038148193	0	4	0	1	2	0
16	ZINC000000839083	2	2	0	0	0	1
17	ZINC000002526389	2	4	1	1	0	1
18	ZINC000028539727	4	4	0	1	3	0
19	ZINC000002526388	2	4	1	1	0	1
20	ZINC000038141997	1	4	0	0	3	1
21	Vemurafenib	1	4	0	1	1	1

a Aqueous-solubility level: 0 (extremely low); 1 (very low, but possible); 2 (low); 3 (good)

b Blood Brain Barrier level: 0 (Very high penetrant); 1 (High); 2 (Medium); 3 (Low); 4 (Undefined)

c Cytochrome P450 2D6 level: 0 (Non-inhibitor); 1 (Inhibitor)

d Hepatotoxicity: 0 (Nontoxic); 1 (Toxic)

e Human-intestinal absorption level: 0 (good); 1 (moderate); 2 (poor); 3 (very poor)

f Plasma Protein Binding: 0 (Absorbent weak); 1 (Absorbent strong)

**Figure 2.**
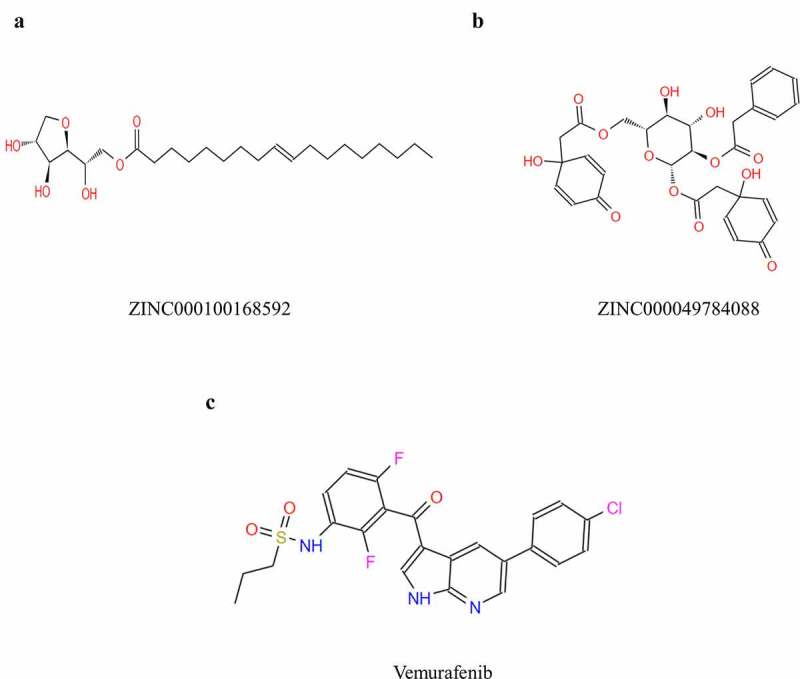
Structures of Vemurafenib and novel compounds selected from virtual screening

Safety was also fully investigated in this study. To examine safety of the selected compounds, different toxicity indicators of the compounds and Vemurafenib, including Ames mutagenicity (AMES), Rodent carcinogenicity (based on the U.S. National Toxicology Program (NTP) dataset) and developmental toxicity potential (DTP) properties, were predicted using TOPKAT module of Discovery Studio 4.5 (Table 3). Results showed that 12 compounds were non-mutagenic. Considering all the results above, ZINC000100168592 and ZINC000049784088 were identified as ideal lead compounds, which were not CYP2D6 inhibitors thereby without hepatotoxicity. Moreover, they were predicted with less ames mutagenicity, rodent carcinogenicity and developmental toxicity potential compared with other compounds, which also strongly suggested their prospective application in drug development. In summary, ZINC000100168592 and ZINC000049784088 were identified as safe drug candidates and selected for following research (Figure 2).

**Table 3.** Toxicities of compounds

Number	Compounds	Mouse NTP^a^		Rat NTP^a^		AMES^b^	DTP^c^
		Female	Male	Female	Male		
1	ZINC000085541163	0.4438	0.3649	0.3071	0.1583	0.0968	0.8155
2	ZINC000017654900	0.5721	0.0048	0.1620	0.5179	0.0000	0.3214
3	ZINC000049784088	0.5414	0.6126	0.2585	0.5119	0.1310	0.5735
4	ZINC000100045922	0.6084	0.5529	0.2799	0.5232	0.5603	0.5334
5	ZINC000040866222	0.5700	0.6130	0.2036	0.1981	0.1534	0.8691
6	ZINC000004098930	0.3688	0.1803	0.1948	0.3959	0.5418	0.7175
7	ZINC000030731451	0.4884	0.5856	0.2788	0.4434	0.7195	0.5720
8	ZINC000015122022	0.2996	0.5764	0.1196	0.1554	0.0002	0.8119
9	ZINC000044352341	0.4447	0.6051	0.1954	0.2056	0.2047	0.8230
10	ZINC000100168592	0.5979	0.6120	0.1388	0.2575	0.0000	0.4667
11	ZINC000021992902	0.5776	0.6142	0.2195	0.4923	0.0008	0.7642
12	ZINC000028115894	0.6187	0.7214	0.5002	0.6762	0.9884	0.7436
13	ZINC000030725991	0.5338	0.6445	0.4743	0.6484	0.9395	0.7590
14	ZINC000029134692	0.7327	0.8030	0.2570	0.5604	0.0225	0.7804
15	ZINC000038148193	0.5782	0.6239	0.4830	0.6617	0.9830	0.7408
16	ZINC000000839083	0.4011	0.5724	0.4773	0.3781	0.4972	0.4622
17	ZINC000002526389	0.3270	0.5091	0.4332	0.4770	0.0001	0.6071
18	ZINC000028539727	0.4132	0.0579	0.2043	0.2855	0.4461	0.4626
19	ZINC000002526388	0.2988	0.4387	0.4216	0.4831	0.0000	0.6178
20	ZINC000038141997	0.7046	0.6141	0.1207	0.4639	0.0000	0.4961
21	Vemurafenib	0.5128	0.6099	0.4866	0.3541	0.7223	0.5099

a < 0.3 (Non-Carcinogen); >0.7 (Carcinogen)

b < 0.3 (Non-Mutagen); >0.7 (Mutagen)

c < 0.3 (Nontoxic); >0.7 (Toxic)

### Analysis of Ligand Binding

**Figure 3.**
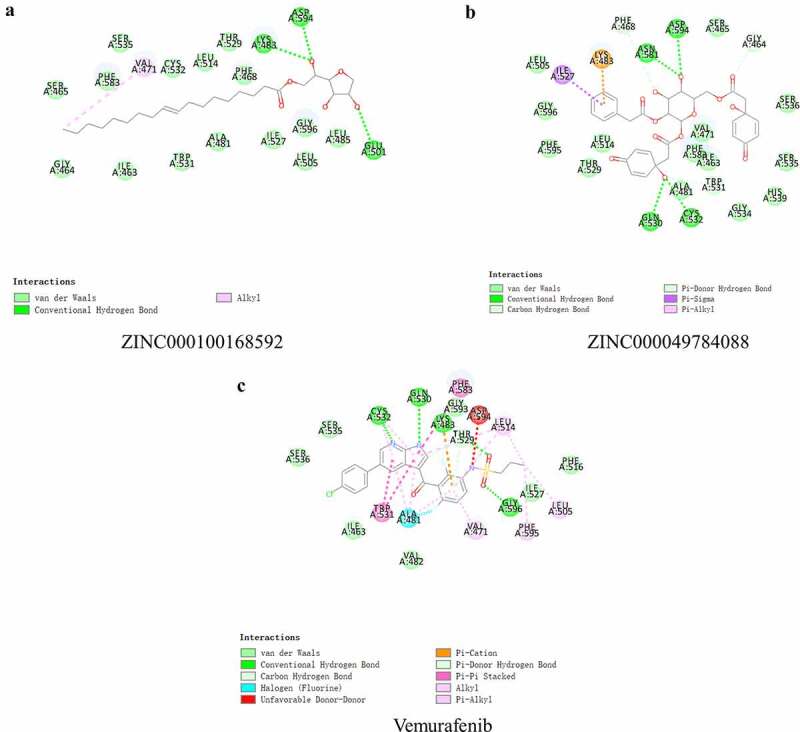
Schematic of intermolecular interaction of the predicted binding modes of (a) ZINC000100168592 with B-RAF(V600E), (b) ZINC000049784088 with B-RAF(V600E), and (c) Vemurafenib with B-RAF(V600E)

**Figure 4.**
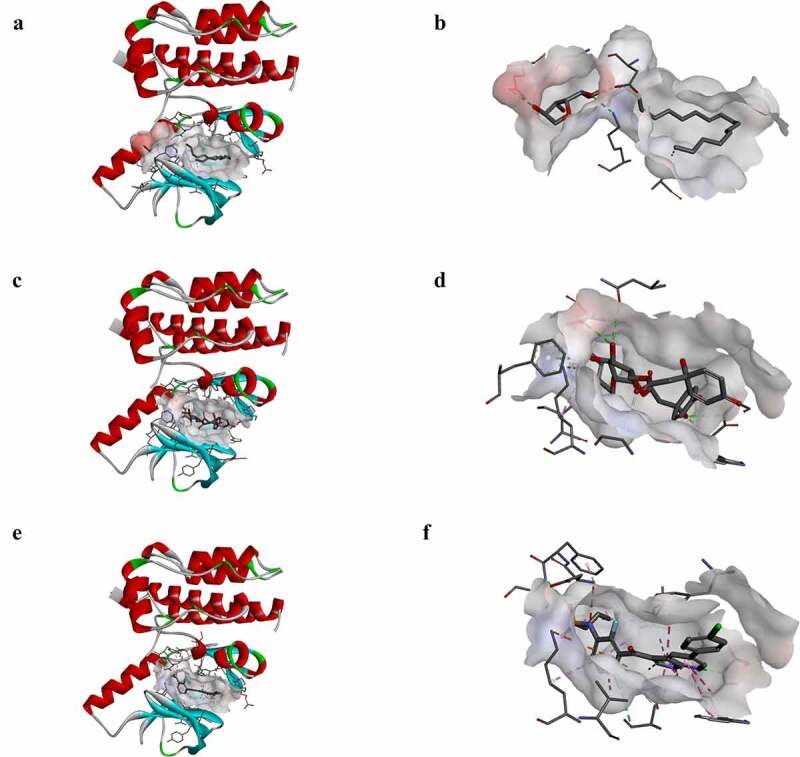
Schematic drawing of interactions between ligands and B-RAF(V600E). The surface of binding areas was added. Blue represents positive charge; red represents negative charge; and ligands are shown in sticks, with the structure around the ligand-receptor junction shown in thinner sticks. (a) ZINC000100168592-B-RAF(V600E) complex. (b) ZINC000049784088-B-RAF(V600E) complex

To study the two selected compounds and Vemurafenib’s ligand-binding mechanisms with B-RAF(V600E), the CDOCKER module was employed. In comparison with ZINC000100168592 and ZINC000049784088, the CDOCKER potential energy of the reference ligand Vemurafenib was significantly higher. These indicated that the binding affinity of B-RAF(V600E) with ZINC000100168592 and ZINC000049784088 was higher than with Vemurafenib (Table 4). Figures 3 and 4 show the**π**-related interactions and hydrogen bonds the structural computation performed. The results of the structural computation study showed that ZINC000049784088 formed 3 pairs of hydrogen bonds with B-RAF(V600E). Also, the complex formed 3 pairs of **π**-related interactions, by the compound itself with LYS483 of B-RAF(V600E), ILE527 of B-RAF(V600E) and LYS483:HZ3 of B-RAF(V600E). Two pairs of hydrogen bonds of ZINC000100168592 with B-RAF(V600E). The complex also formed 1 pair of Alkyl interaction by the compound itself with VAL471 of B-RAF(V600E). The reference Vemurafenib formed 4 hydrogen bonds with B-RAF(V600E), 13 pairs of **π**-related interactions with B-RAF(V600E), 2 pairs of Alkyl interaction and 1 pair of unfavorable donor–donor interaction (Tables 5 and 6).

**Table 4.** CDOCKER interaction energy of compounds with mTORC1

Compounds	CDOCKER Interaction energy (Kcal/mol)
ZINC000049784088	−64.8994
ZINC000100168592	−70.0101
Vemurafenib	−62.5375

**Table 5.** Hydrogen bond interaction parameters for each compound and BRAF(V600E residues

Receptor	Compound	Donor atom	Receptor Atom	Distances (Å)
BRAF(V600E)	Vemurafenib	A:CYS532:N	Molecule:N20	3.24
		A:GLN530:O	Molecule:N18	3.11
		A:LYS483:NZ	Molecule:O5	3.37
		A:GLY596:N	Molecule:O6	3.04
	ZINC000049784088	A:ASN581:OD1	ZINC000049784088:H73	2.87
		A:ASP594:OD2	ZINC000049784088:H73	2.02
		A:GLN530:O	ZINC000049784088:H57	2.23
	ZINC000100168592	A:ASP594:OD1	ZINC000100168592:H67	2.54
		A:LYS483:HZ2	ZINC000100168592:O23	2.02

**Table 6.** π-π interaction, π-Alkyl interaction, Alkyl interaction, π-cation interaction and Unfavorable Doner-Doner interaction, parameters for each compound and mTORC1 residues

Interaction parameters	Receptor	Compound	Donor atom	Receptor Atom	Distances (Å)
π-π interaction	BRAF(V600E)	Vemurafenib	A:TRP531	Molecule	5.87
			A:TRP531	Molecule	4.55
			A:TRP531	Molecule	5.40
			A:PHE583	Molecule	4.90
π-Alkyl interaction		Vemurafenib	A:CYS532	Molecule	4.72
			A:CYS532	Molecule	4.78
			A:ALA481	Molecule	5.10
			A:ALA481	Molecule	3.70
			A:LEU514	Molecule	5.32
			A:ALA481	Molecule	5.33
			A:LEU514	Molecule	5.48
			A:ALY483	Molecule	4.54
			A;phe595	Molecule:C1	4.82
		ZINC000049784088	A:LYS483	ZINC000049784088	4.93
			A:ILE527	ZINC000049784088	4.73
				ZINC000049784088	
Alkyl interaction		Vemurafenib	A:LEU514	Molecule:C1	4.34
			A:LEU505	Molecule:C1	4.62
		ZINC000100168592	A:VAL471	ZINC000100168592:C1	4,5
π-Cation interaction		ZINC000049784088	A:LYS483:HZ3	ZINC000049784088	2.79
Unfavorable Doner-Doner interaction		Vemurafenib	A:ASP594:N	Molecule:N7	3.24

### Molecular Dynamics Simulation

**Figure 5.**
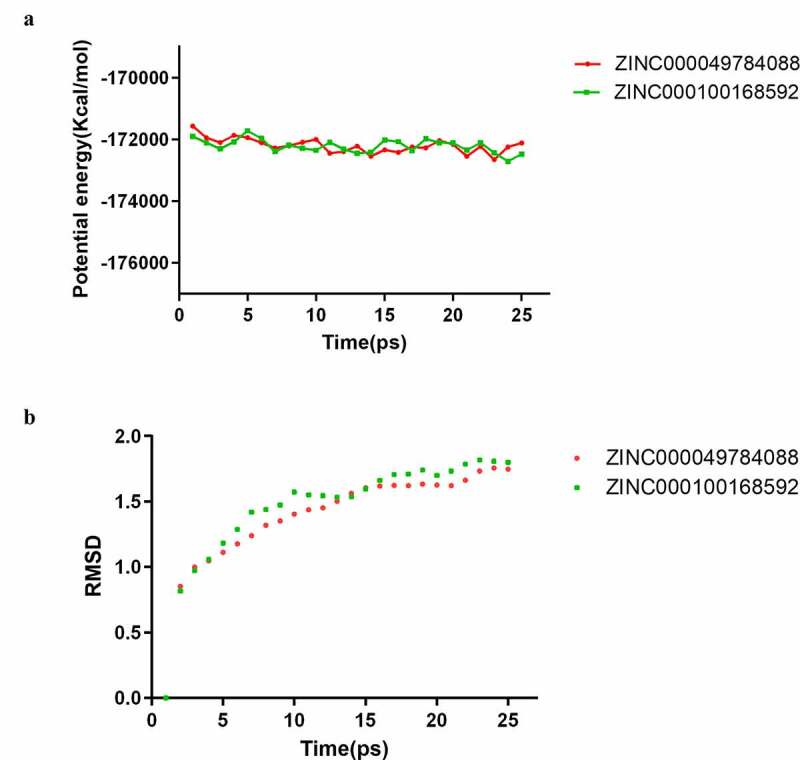
Results of molecular dynamics simulation of the compounds ZINC000100168592 and ZINC000049784088. (a) Potential energy, Average backbone root-mean-square deviation. (b) RMSD, root-mean-square deviation

A molecular dynamics simulation module was built to assess the stability of the ligand- B-RAF(V600E) complexes under natural environmental circumstances. The original conformations were obtained from the CDOCKER module through the molecular docking experiment. Figure 5 shows potential energy and RMSD curves chart of each complex. The time when the trajectories of each complex reached equilibrium was 22.5ps. Potential energy and RMSD of these complexes reached stable state as time goes by. In summary, ZINC000100168592 and ZINC000049784088 could interact with B-RAF(V600E), and the complexes existed stably in the natural environment.

## Discussion

Melanoma is an aggressive skin cancer with significant morbidity and mortality [1]. Oncogenic mutations in B-RAF are found in approximately 40% of patients with cutaneous melanoma and activate the MAP kinase pathway [10]. Nearly 80% of the mutations of B-RAF observed is B-RAF(V600E) mutation [15]. Once the mutation of V600E occurs, it will lead to the continuous abnormal activation of B-RAF, which will lead to the disorder of the regulation of RAS-MAPK signaling pathway and increase the possibility of cancerization. Thus, B-RAF(V600E) plays a vital role in cancer targeted therapy. Despite the great progress in the drug design and development of B-RAF(V600E), only one drug, Vemurafenib, which is the reference drug of this study, received the approval of FDA in 2011 [30]. However, a high incidence of eventual resistance limited the effect of Vemurafenib [31]. Hence, screening more compounds targeting B-RAF(V600E) is urgent.

In order to screen out more potential drug candidates that can inhibit B-RAF(V600E), four modules of the software (Discovery Studio 4.5), including LibDock, ADME/TOPKAT, CDOCKER and Molecular Dynamics Simulation, were employed to screen and analyze the structural biological properties of novel potential compounds, respectively. Molecular conformation, pharmacological properties, binding affinity and stability were also fully analyzed to determine superiority of the selected compounds. In total, 17,799 purchasable, natural, named product molecules were obtained from the ZINC15 database for virtual screening. LibDock score representes the degree of energy optimization and stability of the conformation. Compounds with a high LibDock score indicated that the compound is more geometrically, energetically, and chemically complementary to B-RAF (V600E) than others. After being calculated by the LibDock module of Discovery Studio 4.5, 7763 compounds were identified to have a high binding affinity with B-RAF(V600E). The top 20 natural compounds were selected based on LibDock score and pooled into further study.

In order to analyze the pharmacologic properties of these obtained compounds to reduce the waste of resources and improve the success rate of drug research in later drug development, toxicity predictions and ADME were processed. Results showed that ZINC000100168592 and ZINC000049784088 were identified as ideal lead compounds. They were all soluble in water and also had a good absorption level. Meanwhile, they were non-inhibitors of cytochrome P450 2D6 (CYP2D6), which indicated they did not have hepatotoxicity. Additionally, these three compounds were also predicted with less ames mutagenicity, rodent carcinogenicity and developmental toxicity potential compared with other compounds, which also strongly suggested their prospective application in drug development. On the other hand, the rest drug in the list also had potential application in drug development even though they possessed toxicity, since specific groups and atoms could be added to reduce its toxicity. Considering all the results above, ZINC000100168592 and ZINC000049784088 were selected as ideal lead compounds and further analysis would be carried out.

To conduct more accurate docking analysis of candidate compounds and investigate chemical bonds and ligand-binding mechanisms of B-RAF(V600E) with candidate compounds, the CDOCKER module was employed. Computation results illustrated that CDOCKER the interaction energy of the reference ligand Vemurafenib is higher than ZINC000100168592 and ZINC000049784088, which explains why the binding affinity of ZINC000100168592 and ZINC000049784088 with B-RAF(V600E) is higher than with Vemurafenib. Then, the molecular structure inspection was used to analyze the chemical structure of ZINC000100168592 and ZINC000049784088. Because there were more chemical bonds between these 2 compounds and B-RAF(V600E) in comparison with Vemurafenib, the compounds could bind more stably with B-RAF(V600E), which may strengthen their inhibition of B-RAF(V600E) and thus play a role in improved killing of tumors.

Finally, molecular dynamics simulation was employed to evaluate whether they can maintain stability in the natural environment. The calculation results of potential energy and RMSD of these ligand-B-RAF(V600E) complexes illustrated that the time at which the trajectories of complexes reached equilibrium is 22.5 ps. With time passing, potential energy and RMSD of these complexes reached a stable state which manifested that these two complexes could keep stable in the natural environment. Prospectively, the refinement and modification of the drugs could be proceeded in order to make ligands and receptors bind in a more stable state on the basis of the results above. Because of the provision of high stabilization and binding affinity of the selected drugs in this research, development of inhibitive durg of B-RAF(V600E) was strengthened.

In spite of the fact that overall research on oncology drugs has not progressed well, development and design of them are still a research hotspot. This study indicated that screening more ideal lead compounds is the most important in current development and design of oncology drugs. In order to screen the structure of novel natural compounds and select potential drug candidates, numerous modules of the software (Discovery Studio 4.5) were employed in this study. Results demonstrated that the binding affinity, molecule conformation and stability of each selected compound were better than the reference compound Vemurafenib, besides, these two compounds may have huge therapeutic potential in B-RAF(V600E)-related cancer.

In summary, this study aimed to find more potential drug candidates which can inhibit B-RAF(V600E) form a natural compounds database. Even though this study was conducted by elaborate design and precise measurements was performed, we still admitted that there were still some limitations in this study. Without being improved and refined, no drug can be marketed. To make these two compounds more perfect as drug candidates, some groups and atoms which can influence the pharmacological properties of the drugs are needed to modify. More experiments need to be performed to validate our results and more indicators regarding to drugs safety, such as MTD (Maximum Tolerated Dosage) and AB(Aerobic Biodegradability) should also be assessed in our future study. These limitations are directions and focus of our further study.

## Conclusion

This study performed a series of computer-aided structural and chemical analysis technology (including virtual screening, molecule docking, ADME, toxicity prediction) to screen and identify the ideal-leading compound with functions to inhibit B-RAF(V600E). Two compounds, ZINC000100168592 and ZINC000049784088, were predicted as potential inhibitors targeting B-RAF(V600E). The experiments carried out by Discovery Studio4.5 confirmed that these two compounds could bind tightly with B-RAF(V600E) in the natural environments simulated by the MD simulation module. In spite of the limitations, this study lays the groundwork for future clinical trials of these two compounds. Moreover, these novel natural compounds with structural modifications can be potential contributors for leads in further rational drug design for targeting B-RAF(V600E).

## Data Availability

The datasets used and/or analyzed during the current study are available from the corresponding author on reasonable request

## Abbreviation

Central Nervous System, CNS;

mitogen-activated protein kinase, MAPK;

progression-free survival, PFS;

overall survival, OS;

metastatic castration-resistant prostate cancer, mCRPC;

Absorption, distribution, metabolic, excretion, ADME;

Discovery Studio 4.5 software, DS4.5;

Natural Products, NP;

University of California, San Francisco, UCSF;

Protein data bank, PDB;

root mean square, RSM;

toxicity prediction by Computer assistive technology, TOPKAT;

blood–brain barrier, BBB;

cytochrome P4502D6, CYP2D6;

Plasma protein, PPB;

## References

[1] Pavri SN, Clune J, Ariyan S, et al. Malignant Melanoma: beyond the Basics. Plast Reconstr Surg. 2016;138(2):330e–40e. 10.1097/PRS.0000000000002367 27465194

[2] Wan M, Zhuang B, Dai X, et al. A new metabolic signature contributes to disease progression and predicts worse survival in melanoma. Bioengineered. 2020;11(1):1099–1111. 33084485 10.1080/21655979.2020.1822714 PMC8291831

[3] Pasquali S, Hadjinicolaou AV, Chiarion Sileni V, et al. Systemic treatments for metastatic cutaneous melanoma. Cochrane Database Syst Rev. 2018;2(2):Cd011123. 29405038 10.1002/14651858.CD011123.pub2 PMC6491081

[4] Cheng PF. Medical bioinformatics in melanoma. Curr Opin Oncol. 2018;30(2):113–117. 29227308 10.1097/CCO.0000000000000428

[5] Kızılbey K, Mansuroğlu B, Derman S, et al. An In vivo study: adjuvant activity of poly-n-vinyl-2-pyrrolidone-co-acrylic acid on immune responses against Melanoma synthetic peptide. Bioengineered. 2018;9(1):134–143. 28910565 10.1080/21655979.2017.1373529 PMC5972930

[6] Abbas O, Miller DD, Bhawan J. Cutaneous malignant melanoma: update on diagnostic and prognostic biomarkers. Am J Dermatopathol. 2014;36(5):363–379. 24803061 10.1097/DAD.0b013e31828a2ec5

[7] Cuevas LM, Daud AI. Immunotherapy for melanoma. Semin Cutan Med Surg. 2018;37(2):127–131. 30040090 10.12788/j.sder.2018.028

[8] Cohen JV, Buchbinder EI. The Evolution of Adjuvant Therapy for Melanoma. Curr Oncol Rep. 2019;21(12):106. 31768772 10.1007/s11912-019-0858-3

[9] Quaglino P, Fava P, Brizio M, et al. Anti-BRAF/anti-MEK targeted therapies for metastatic melanoma patients during the COVID-19 outbreak: experience from an Italian skin cancer unit. Future Oncol. 2021;17(7):759–761. 33533662 10.2217/fon-2020-0997 PMC7874884

[10] Degirmenci U, Wang M, Hu J. Targeting Aberrant RAS/RAF/MEK/ERK Signaling for Cancer Therapy. Cells. 2020;9(1):1. 10.3390/cells9010198 PMC7017232 31941155

[11] Rabbie R, Ferguson P, Molina-Aguilar C, et al. Melanoma subtypes: genomic profiles, prognostic molecular markers and therapeutic possibilities. J Pathol. 2019;247(5):539–551. 30511391 10.1002/path.5213 PMC6492003

[12] Sun J, Carr MJ, Khushalani NI. Principles of Targeted Therapy for Melanoma. Surg Clin North Am. 2020;100(1):175–188. 31753111 10.1016/j.suc.2019.09.013

[13] Da R, Wang M, Jiang H, et al. BRAF (AMP) Frequently Co-occurs With IDH1/2, TP53, and ATRX Mutations in Adult Patients With Gliomas and Is Associated With Poorer Survival Than That of Patients Harboring BRAF (V600E). Front Oncol. 2020;10:531968. 33489866 10.3389/fonc.2020.531968 PMC7817544

[14] Dulgar O, Kutuk T, Eroglu Z. Mechanisms of Resistance to BRAF-Targeted Melanoma Therapies. Am J Clin Dermatol. 2021;22(1):1–10. 33368052 10.1007/s40257-020-00572-6

[15] Flaherty KT. BRAF inhibitors and melanoma. Cancer J. 2011;17(6):505–511. 22157295 10.1097/PPO.0b013e31823e5357

[16] Patel H, Yacoub N, Mishra R, et al. Current Advances in the Treatment of BRAF-Mutant Melanoma. Cancers (Basel). 2020;12(2):2. 10.3390/cancers12020482 PMC7072236 32092958

[17] Gutzmer R, Stroyakovskiy D, Gogas H, et al. Atezolizumab, vemurafenib, and cobimetinib as first-line treatment for unresectable advanced BRAF(V600) mutation-positive melanoma (IMspire150): primary analysis of the randomised, double-blind, placebo-controlled, phase 3 trial. Lancet. 2020;395(10240):1835–1844. 32534646 10.1016/S0140-6736(20)30934-X

[18] Khaddour K, Kurn H, Zito PM. Vemurafenib. StatPearls. Treasure Island (FL): StatPearls Publishing Copyright © 2021. StatPearls Publishing LLC; 2021.

[19] Cragg GM. Natural product drug discovery and development: the United States National Cancer Institute role. P R Health Sci J. 2002;21(2):97–111. 12166031

[20] Cragg GM, Newman DJ. Natural products: a continuing source of novel drug leads. Biochim Biophys Acta. 2013;1830(6):3670–3695. 23428572 10.1016/j.bbagen.2013.02.008 PMC3672862

[21] How CW, Ong YS, Low SS, et al. How far have we explored fungi to fight cancer? Semin Cancer Biol. 2021. DOI:10.1016/j.semcancer.2021.03.009 33737109

[22] Zhong S, Li W, Bai Y, et al. Computational study on new natural compound agonists of stimulator of interferon genes (STING). PLoS One. 2019;14(5):e0216678. 31120925 10.1371/journal.pone.0216678 PMC6532845

[23] Shin Low S, Pan Y, Ji D, et al. Smartphone-based portable electrochemical biosensing system for detection of circulating microRNA-21 in saliva as a proof-of-concept. Sens Actuators B Chem. 2020;308:127718.

[24] Sterling T, Irwin JJ. ZINC 15--Ligand Discovery for Everyone. J Chem Inf Model. 2015;55(11):2324–2337. 26479676 10.1021/acs.jcim.5b00559 PMC4658288

[25] Rao SN, Head MS, Kulkarni A, et al. Validation studies of the site-directed docking program LibDock. J Chem Inf Model. 2007;47(6):2159–2171. 17985863 10.1021/ci6004299

[26] Brooks BR, Brooks CL 3rd, Mackerell AD Jr., et al. CHARMM: the biomolecular simulation program. J Comput Chem. 2009;30(10):1545–1614. 19444816 10.1002/jcc.21287 PMC2810661

[27] Han Y, Zhang J, Hu CQ, et al. In silico ADME and Toxicity Prediction of Ceftazidime and Its Impurities. Front Pharmacol. 2019;10:434. 31068821 10.3389/fphar.2019.00434 PMC6491819

[28] Gagnon JK, Law SM, Brooks CL 3rd. Flexible CDOCKER: development and application of a pseudo-explicit structure-based docking method within CHARMM. J Comput Chem. 2016;37(8):753–762. 26691274 10.1002/jcc.24259 PMC4776757

[29] Tabassum H, IZ A. Molecular Docking and Dynamics Simulation Analysis of Thymoquinone and Thymol Compounds from Nigella sativa L. that Inhibits P38 Protein: probable Remedies for Hepatocellular Carcinoma. Med Chem. 2020;16(3):350–357. 31038073 10.2174/1573406415666190416165732

[30] Fiskus W, Mitsiades N. B-Raf Inhibition in the Clinic: present and Future. Annu Rev Med. 2016;67(1):29–43. 26768236 10.1146/annurev-med-090514-030732

[31] Broman KK, Dossett LA, Sun J, et al. Update on BRAF and MEK inhibition for treatment of melanoma in metastatic, unresectable, and adjuvant settings. Expert Opin Drug Saf. 2019;18(5):381–392. 30977681 10.1080/14740338.2019.1607289

